# Challenges in the determination of total vitamin B12 by cyanidation conversion: insights from stable isotope dilution assays

**DOI:** 10.1007/s00216-023-04860-y

**Published:** 2023-07-19

**Authors:** Mengle Wang, Kathrin Schuster, Stefan Asam, Michael Rychlik

**Affiliations:** Chair of Analytical Food Chemistry, Department of Life Science Engineering, School of Life Sciences, Technical University of Munich, Maximus-von-Imhof-Forum 2, 85354 Freising, Germany

**Keywords:** Cyanidation, Conversion rate, Cobalamin, Vitamin B12, Stable isotope dilution assay

## Abstract

Previous methods for vitamin B12 (B12) analysis have extensively used cyanidation conversion with the intention of converting all cobalamins to cyanocobalamin (CNCbl) for total B12 determination. This approach has been favored for its advantages in reducing the number of analytes, increasing analyte concentration, and improving analyte stability. However, the present study revealed underlying limitations associated with this approach. First, a stable isotope dilution assay (SIDA) determining total B12 as CNCbl after cyanidation conversion (conversion SIDA method) was developed. Method validation demonstrated good sensitivity, recovery, accuracy, and reproducibility for the target analyte CNCbl. However, subsequent application of the conversion method to real meat samples showed incomplete conversions of cobalamins. These inconsistencies revealed day-to-day variability and reliability challenges associated with the cyanidation process. It was not possible to identify this issue during method validation as CNCbl was spiked as the sole analyte and it requires no further cyanidation conversion. The application of LC-MS/MS enabled the detection of trace amounts of unconverted cobalamins. Nevertheless, this approach remains restricted by instrument sensitivity and stability as well as the performance of immunoaffinity purification for different vitamers. Further development of a reliable monitoring method is a prerequisite for further optimization of the cyanidation process. However, significant improvements of analytical instrumentation in terms of sensitivity and stability are required to overcome the current limitations.

**Graphical abstract:**

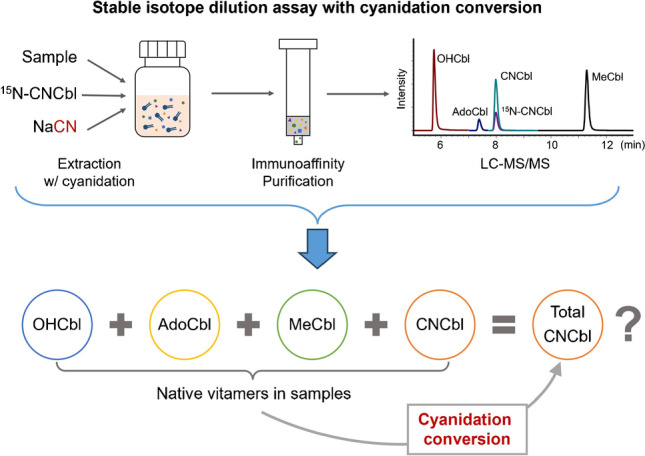

**Supplementary Information:**

The online version contains supplementary material available at 10.1007/s00216-023-04860-y.

## Introduction

**Fig. 1:**
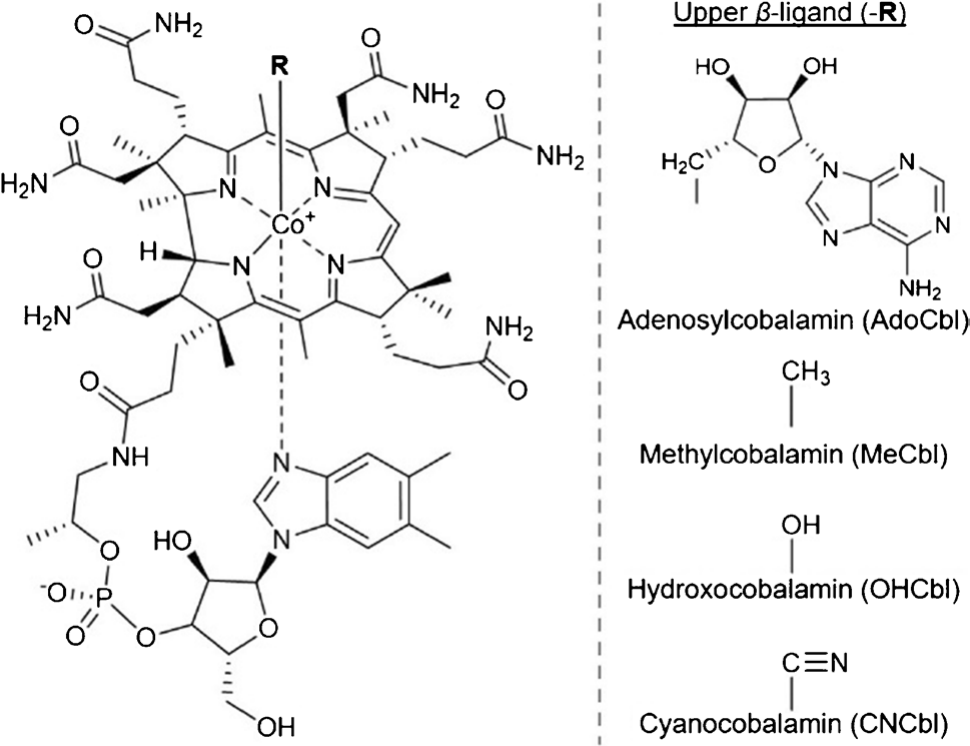
Structure of vitamin B12. The upper ligand (R) differs to form individual cobalamins with corresponding names and abbreviations given below

Vitamin B12 (B12) represents a family of compounds called cobalamins, which possess the same core structure but different upper *β*-ligands (Fig. 1). In nature, the most common cobalamins are adenosylcobalamin (AdoCbl), methylcobalamin (MeCbl), hydroxocobalamin (OHCbl), and cyanocobalamin (CNCbl).

B12 is well-known for its biological significance in maintaining normal human physiology. At the molecular level, MeCbl and AdoCbl are required as cofactors, respectively, for cytosolic methionine synthase (E.C. 2.1.1.13) and mitochondrial methylmalonyl-CoA mutase (E.C. 5.4.99.2) [1, 2]. The corresponding enzymatic reactions are crucial for various metabolic activities, such as DNA synthesis and repair [3] and breakdown of branched-chain amino acids and odd-chain fatty acids [3, 4]. OHCbl, CNCbl, and other cobalamins play their biological roles after being converted into these two coenzymatically active forms in the cells.

A special attribute of B12 is its exclusive origin of certain prokaryotes [5]. As humans are not able to synthesize B12 in vivo and predominantly rely on B12 from diets, the analysis of B12 in foods is essential for identifying B12-rich dietary sources as well as for monitoring the daily intakes.

The analysis of B12 in foods is a challenging task due to low concentrations of natural vitamers in non-supplemented foods, chemical instability of cobalamins, and potential presence of a wide range of structural analogs in the matrices [6, 7]. Until the recent breakthrough on preparation of ^15^N-labeled cobalamins via biosynthesis [8] and further development of the first multiple stable isotope dilution assay (SIDA) that simultaneously determines the four natural vitamers [7], the quantification of B12 in foods has been relying on methods mainly applying microbiological assays [9, 10], (U)HPLC-UV [11–17], and occasionally LC-ICP-MS [18].

To ease the analytical difficulties with limitations on sensitivity or specificity, previous applications often make use of cyanidation conversion of native B12 vitamers into CNCbl during sample preparation. The total B12 contents of the samples are then determined as total CNCbl. The cyanidation practice is beneficial for B12 analysis due to improved overall analyte stability and increased overall analyte concentrations. Since CNCbl was the sole analytical target after cyanidation conversion, the corresponding methods were merely validated for the determination of CNCbl in respective food matrices. The performance of the cyanidation conversion, which is highly relevant to the obtained total B12 contents, however, was generally neglected during the validation and further applications of the methods. This fact is rather concerning considering the popularity of cyanidation conversion-based methods for B12 quantification. Further investigations are thus highly required to verify the correctness of total B12 contents obtained with the application of cyanidation conversion.

The present study aimed to develop and validate a conversion SIDA method that determines total B12 as CNCbl after cyanidation conversion and to further compare the developed method with a previously developed multi-SIDA method targeting individual native vitamers (hereafter referred as native SIDA method). By doing so, the present study intended to investigate the suitability and reliability of cyanidation conversion for B12 analysis in foods. A direct comparison between corresponding results from the two methods is feasible due to the application of the same principle for quantification, i.e., SIDA.

## Materials and methods

### Chemicals and materials

Analytical standards of CNCbl, OHCbl·HCl, AdoCbl, and MeCbl were obtained from Sigma-Aldrich (Steinheim, Germany). [^15^N_13_]-CNCbl was prepared in-house as previously described [8]. In general, ^15^N-labeled cobalamins were prepared via biosynthesis using *Propionibacterium freudenreichii*. The labeled cobalamins were either directly isolated from the cell extracts or further treated with additional chemical modification steps to obtain different vitamers. Papain from *papaya latex* (EC 3.4.22.2, cat no. P4762, ≥ 10 units/mg protein), *α*-amylase from *Aspergillus oryzae* (EC 3.2.1.1, cat. no. 10065, ~30 U/mg), and soybean flour (type I, cat. no. S9633) were purchased from Sigma-Aldrich (Steinheim, Germany).

Sodium cyanide (≥ 97%) and sodium acetate trihydrate (≥ 99.0%) were purchased from Sigma-Aldrich (Steinheim, Germany). Water (LC-MS and HPLC grade) and methanol (HPLC grade) were obtained from Th. Geyer (Renningen, Germany). Acetic acid for LC-MS (≥ 99.8%), glacial acetic acid (HPLC grade), and ammonium acetate for LC-MS (≥ 99.0%) were purchased from VWR International (Ismaning, Germany). Methanol (LC-MS grade) was purchased from Honeywell (Seelze, Germany).

### Samples

For the development of the conversion SIDA method, a fresh pork fillet sample (~300 g) was purchased from a local butcher shop (Freising, Germany) in minced form. The sample was lyophilized (Alpha 1-2 LDplus, Martin Christ Gefriertrocknungsanlagen, Osterode am Harz, Germany), finely ground (EGK 200, Rommelsbacher, Dinkelsbühl, Germany), thoroughly homogenized, and stored at −20 °C in the dark until further analysis.

Meat samples (pork fillet, beef fillet, lamb fillet, and chicken breast) that have been previously analyzed by the native SIDA method for individual cobalamins [7] were again analyzed using the conversion SIDA method for total B12 contents. These samples were stored at −20 °C in the dark.

### Sample preparation of conversion SIDA method

The extraction and purification were developed based on the published native SIDA method [7] as well as previous cyanidation conversion-based methods [12–14, 19]. During method development, various sample preparation methods were tested on a pork fillet sample in order to investigate and optimize the conversion process. The details of each method are described below. Table 1 summarizes the differences between the methods.

**Table 1:** Summary of different conditions between extraction methods A–D

	Extraction steps			
Method	Stirring (20 min, RT)	Incubation (1 h, 37 °C)	Heating in boiling water bath	2nd cyanidation after paper filtration
A	Subdued light	Subdued light	Subdued light, 10 min	Not applied
B	Normal light	Normal light	Normal light, 10 min	Not applied
C	Normal light	Normal light	Normal light, 30 min	Not applied
D	Normal light	Normal light	Normal light, 30 min	Addition of 100 μL of 1% NaCN (w/v);10 min shaking under normal light

#### Method A

One gram of lyophilized and homogenized meat sample, 5 mg of papain, and 5 mg of *α*-amylase were weighed into a 50-mL amber extraction vial (Duran, Mainz, Germany). As internal standard, [^15^N_13_]-CNCbl was added in amounts based on the expected total contents of cobalamins in the samples to fall inside the calibration range. Sodium cyanide solution (500 μL, 1%, w/v) and extraction buffer (25 mL, 50 mM sodium acetate buffer, pH = 4) were added to the sample. The sample mixture was vortexed and mixed using a magnetic stirrer (IKA, RO 15 power, Staufen, Germany) at room temperature (RT) for 20 min before being incubated in a shaking water bath (GFL 1092, Burgwedel, Germany) at 37 °C for 1 h. Afterwards, the sample homogenate was heated in a boiling water bath for 10 min, cooled in an ice water bath, and transferred to a 50-mL amber centrifuge tube. The residue in the extraction vial was rinsed with 5 mL of extraction buffer and was further incorporated into the centrifuge tube. The sample was then centrifuged (Eppendorf 5810R, Hamburg, Germany) for 20 min (3220 *× g*, RT) and the supernatant was collected and paper filtered (Whatman 597^1/2^) for immunoaffinity purification.

The samples were purified using immunoaffinity columns (EASI-Extract, Vitamin B12, R-Biopharm, Glasgow, UK) following the same procedures of the native SIDA method [7]. After passing the sample filtrate through the column, the column was washed with 10 mL of water. Afterwards, an elution step using 2 mL of methanol with back flushing was conducted twice. The eluate was dried at 40 °C under a stream of nitrogen in an evaporator system (EC2, VLM, Bielefeld, Germany). The sample was reconstituted in 300 μL of LC-MS water and membrane filtered (PVDF, Ø13 mm, 0.22 μm, Ahlstrom Munksjö, Helsinki, Finland) before LC-MS/MS analysis. All steps were performed under subdued light conditions.

#### Method B

The same steps from method A were adopted under different light conditions. Clear extraction vials and centrifuge tubes instead of the amber ones were used for extraction. The whole extraction was performed under normal laboratory light conditions. After extraction, the immunoaffinity purification was conducted under subdued light.

#### Method C

The procedures and light conditions were the same as that of method B except that the sample homogenate was heated in the boiling water bath for 30 min (only 10 min for method B) after the incubation.

#### Method D

On the basis of method C, a second cyanidation step was incorporated. After paper filtration, 100 μL of sodium cyanide solution (1%, w/v) was added to the filtrate, and the filtrate was then shaken horizontally at 300 rpm (Shaker KL 2, Edmund Bühler, Bodelshausen, Germany) for 10 min under normal laboratory light conditions. Subsequently, the filtrate went through immunoaffinity purification under subdued light conditions.

### Preparation and concentration determination of standard solutions

The preparation of standard solutions was conducted following the procedures previously developed for the native SIDA method [7] with slight modifications. Briefly, a stock solution of unlabeled CNCbl was prepared by dissolving 1 mg of the reference compound in 10 mL of water (~ 0.1 mg/mL). The unlabeled CNCbl stock solution was prepared monthly and was divided into small aliquots for storage at −20 °C in the dark. A freshly thawed aliquot was used for each extraction. The accurate concentration of the CNCbl stock solution was determined daily by UV spectroscopy after a 4-fold dilution with water. A stock solution of isotopically labeled [^15^N_13_]-CNCbl (∼10 μg/mL) was prepared separately in water. For sample preparation, the labeled stock solution was further diluted to obtain working solutions with concentrations suitable for spiking purposes in the range of 0.01 and 0.5 μg/mL. To determine the accurate concentrations of [^15^N_13_]-CNCbl used for each extraction, a calibrator solution comprising unlabeled CNCbl (∼50-100 ng/mL) and labeled [^15^N_13_]-CNCbl (∼50-100 ng/mL) was routinely prepared and measured by LC-MS/MS. To obtain the calibrator, the CNCbl stock solution was diluted and further mixed with [^15^N_13_]-CNCbl. Based on the LC-MS/MS response curve of CNCbl, the concentrations of [^15^N_13_]-CNCbl were calculated from the known concentrations of CNCbl previously determined by UV spectroscopy.

For the determination of conversion rate, stock solutions of OHCbl, AdoCbl, and MeCbl were prepared by dissolving 1 mg of respective reference compound in 10 mL of water. The accurate concentrations of the stock solutions were determined by HPLC-DAD using CNCbl as internal standard as previously reported [7]. Briefly, a mixed standard solution consisting of the four cobalamins, prepared by mixing water (150 μL), CNCbl (100 μL of ~0.1 mg/mL), OHCbl (250 μL of ~0.1 mg/mL), AdoCbl (250 μL of ~0.1 mg/mL), and MeCbl (250 μL of ~0.1 mg/mL), was measured using HPLC-DAD. The accurate concentration of CNCbl was beforehand obtained from UV spectroscopy measurements. The concentrations of the other three cobalamins were calculated from the known concentration of CNCbl based on the previously established HPLC-DAD response functions (see Electronic Supplementary Material Table S1). For spiking purposes, a working solution comprising OHCbl, AdoCbl, and MeCbl (∼200 ng/mL, respectively) was prepared by diluting and mixing the stock solutions.

All standard solutions were stored at −20 °C protected from light. The CNCbl stock solutions were prepared monthly and the stock solutions of the other cobalamins were used within 2 months. For storage, the stock solutions were pre-divided into 1 mL aliquots. For each extraction, freshly thawed aliquots were used.

### Instrumental conditions

#### UV spectroscopy

A Genesys 10S UV-VIS spectrophotometer (Thermo Fischer Scientific, Madison, USA) was used for the absorption measurements at 361 nm. Disposable cuvettes (optical path length of 1 cm, PMMA, VWR, Ismaning, Germany) were used, and LC-MS water was measured as blank. Further details were reported previously [7].

#### HPLC-DAD

The HPLC-DAD system (Shimadzu, Kyoto, Japan) consisted of an auto-sampler (SIL-20A), a liquid chromatograph (LC-20AD), and a diode array detector (SPD-M20A). As stationary phase, a Triart C18 column (150 × 3.0 mm, 3 μm, YMC, Dinslaken, Germany) was utilized for chromatographic separation. A 50-mM ammonium acetate buffer (pH = 4) and 100% methanol served as mobile phases A and B, respectively. Further details on the gradient and instrumental parameters were described earlier [7].

#### LC-MS/MS

The LC-MS/MS measurements were conducted on a Nexera X2 UHPLC (Shimadzu, Kyoto, Japan) coupled to a triple quadrupole mass spectrometer (LC-MS 8050, Shimadzu, Kyoto, Japan) under positive electrospray ionization (ESI) mode. A Hydrosphere C18 column (150 × 3.0 mm, 3 μm, YMC, Dinslaken, Germany) with a C18 pre-column (4 × 2.0 mm, Phenomenex, Aschaffenburg, Germany) served as stationary phase for chromatographic separation. Mobile phases A and B were 0.1% acetic acid in water and 100% methanol, respectively. The acquisition was performed in scheduled multiple reaction monitoring (MRM) mode. The gradient and instrumental conditions were the same as previously reported for the native SIDA method [7]. The detailed MRM parameters are listed in Table S2, Electronic Supplementary Material. The MRM transitions of OHCbl, AdoCbl, and MeCbl were monitored for the detection of unconverted cobalamins. LabSolutions software (Shimadzu, Kyoto, Japan) was used for system control, data acquisition, and data analysis.

### LC-MS/MS calibration and quantitation of the conversion SIDA method

After cyanidation conversion, cobalamins in the samples were quantified as total CNCbl using [^15^N_13_]-CNCbl as internal standard. The previously established LC-MS/MS response curve for CNCbl (*y* = 0.001*x*^2^ + 1.0643*x* − 0.0276, *R*^2^ = 0.9999) was used for quantification [7]. The *Y*-axis is the peak area ratio [*A*(CNCbl)/*A*([^15^N_13_]-CNCbl)], and the *X*-axis is the molar ratio [n(CNCbl)/n([^15^N_13_]-CNCbl)] ranging between 0.01 and 115.61. The contents of CNCbl in all samples were calculated based on the LC-MS/MS response curve. The curve was mathematically resolved for n(CNCbl). The results given in the present study are based on dry matter.

### Method validation of the conversion SIDA method

#### LOD and LOQ

Limits of detection and quantification (LOD, LOQ) of the conversion SIDA method for CNCbl were determined according to Vogelgesang and Hädrich [20] in combination with the surrogate analyte approach [21]. The purchased soybean flour was used as the surrogate matrix, and the self-prepared [^15^N_13_]-CNCbl was used as the surrogate analyte for CNCbl. Four different levels (0.2, 0.7, 1.4, and 2.1 ng/g) of the surrogate analyte (i.e., [^15^N_13_]-CNCbl) were spiked to the soybean flour, and each spiking level was prepared in quadruplicate. For quantification, CNCbl was added as internal standard during sample preparation.

Based on previous LC-MS/MS measurements used for establishing the LC-MS/MS response curve of CNCbl for the native SIDA method [7], a reverse LC-MS/MS response curve was obtained by plotting peak area ratios [*A*([^15^N_13_]-CNCbl)/*A*(CNCbl)] against the molar ratios [n([^15^N_13_]-CNCbl)/n(CNCbl)] for the quantification of [^15^N_13_]-CNCbl. The reverse LC-MS/MS response curve (*y* = 1.0088*x* − 0.014, *R*^2^ = 0.9999) was linear in molar ratios between 0.043 and 8.65 with *Y*-axis being peak area ratio [*A*([^15^N_13_]-CNCbl)/*A*(CNCbl)] and *X*-axis being the molar ratio [n([^15^N_13_]-CNCbl)/n(CNCbl)]. The linearity was confirmed by the Mandel’s fitting test [22]. In this way, the LOD and LOQ of the conversion SIDA method were calculated from [^15^N_13_]-CNCbl instead of CNCbl using the reverse LC-MS/MS response curve.

#### Recovery

The recoveries of CNCbl were determined by analyzing a pork fillet sample spiked with CNCbl at three different levels (7.5, 37.6, 74.7 ng/g). Each spiking level was prepared in triplicate. The pork fillet sample was analyzed applying the conversion SIDA method before and after spiking. The recoveries were calculated for CNCbl using the following equation:

$$\textrm{Recovery}\ \left(\%\right)=\frac{\textrm{found}\ \textrm{amount}\ \textrm{of}\ \textrm{total}\ \textrm{CNCbl}\ \left(\textrm{ng}/\textrm{g}\right)-\textrm{endogenous}\ \textrm{amount}\ \textrm{of}\ \textrm{total}\ \textrm{CNCbl}\ \left(\textrm{ng}/\textrm{g}\right)}{\textrm{spiked}\ \textrm{amount}\ \left(\textrm{ng}/\textrm{g}\right)} \times 100\%$$

#### Precision

Inter-injection (*n* = 6), intra-day (*n* = 4), and inter-day precisions (*n* = 3, quadruplicate analysis each week for 3 weeks) were determined by analyzing a pork fillet sample containing all four cobalamins using the conversion SIDA method.

### Determination of conversion rate

A pork fillet sample was spiked with OHCbl (35.0 ng/g), AdoCbl (27.3 ng/g), and MeCbl (39.5 ng/g). The total CNCbl contents were analyzed using the conversion SIDA method before and after spiking. The spiked amount of each cobalamin was determined by HPLC-DAD as described in the “HPLC-DAD” section. The conversion rate was calculated as follows:

$$\textrm{Conversion}\ \textrm{rate}\ \left(\%\right)=\frac{\textrm{total}\ \textrm{CNCbl}\ \textrm{of}\ \textrm{spiked}\ \textrm{sample}\ \left(\textrm{ng}/\textrm{g}\right)-\textrm{total}\ \textrm{CNCbl}\ \textrm{of}\ \textrm{original}\ \textrm{sample}\ \left(\textrm{ng}/\textrm{g}\right)\ }{\textrm{total}\ \textrm{spiked}\ \textrm{amount}\ \textrm{calculated}\ \textrm{as}\ \textrm{CNCbl}\ \textrm{equivalent}\ \left(\textrm{ng}/\textrm{g}\right)}\times 100\%$$

## Results and discussion

### Sample preparation of the conversion SIDA method

The sample preparation of the conversion SIDA method was developed and optimized aiming at achieving complete conversions of all native cobalamins into the cyano-form, a prerequisite for accurate quantification of total B12 as CNCbl. The conversions of cobalamins from different extractions were monitored by determining the contents of total B12 in the pork fillet sample applying SIDA as well as observing the peaks of unconverted OHCbl, AdoCbl, and MeCbl in the LC-MS/MS chromatograms.

In preliminary experiments, cyanide was first used in amounts (250 μL of 1% NaCN solution (w/v)) equivalent to a literature value [14] for light-protected extractions in amber vials following the same procedures of the native SIDA method [7]. The obtained LC-MS/MS chromatograms clearly revealed incomplete conversions of AdoCbl and MeCbl in the sample solutions (data not shown). In order to minimize photo-degradation of cobalamins during extraction, sample preparation of the native SIDA method was conducted under light-protected conditions. Subdued light conditions were as well commonly required by previous cyanidation-based methods [12–14, 19] considering the photo-liability of cobalamins. However, the cobalt–carbon bonds in MeCbl and AdoCbl were found to be rather stable in the dark under neutral, diluted acidic, and alkaline conditions [23]. In a previous study [24], MeCbl remained unchanged in the dark in the presence of excess potassium cyanide (molar ratio of 50:1). To facilitate the ligand substitution of AdoCbl and MeCbl via OHCbl, the form that readily reacts with cyanide to generate CNCbl, light exposure might be necessary for sample extraction. It is also possible that the amount of cyanide first tested was not adequate for converting all cobalamins in the sample. In addition, the duration of 10 min for heating in the boiling water bath, which was adopted from the native SIDA method, was shorter than the common duration of 30 min used in the literature [13, 14]. A prolonged boiling time might be needed to achieve complete conversions. Therefore, the sample preparation method was further optimized based on the following parameters: (i) application and duration of light exposure, (ii) duration of boiling, and (iii) the amount of cyanide added. Apart from the performances on conversion, these parameters were optimized taking additional factors into consideration. First, the increase on cyanide amount used for extraction leads to increased safety concerns of the experiments. Moreover, excessive use of cyanide might generate dicyanocobalamin (di-CNCbl) instead of CNCbl in the sample solution [24]. The prolonged heating time and light exposure might be beneficial for the conversion but generally also increase the risk of degradation of all cobalamins.

Double amount of cyanide (500 μL of 1% NaCN solution (w/v)) was first tested for the extraction of a pork fillet sample under light-protected conditions (method A). The increase on cyanide amount did not lead to complete conversions, and a total CNCbl content of 13.1 ng/g was obtained. When the same extraction procedures were performed under normal laboratory light conditions (method B), the obtained total CNCbl content increased to 24.1 ng/g, indicating that light exposure might be necessary for a complete and timely conversion. With a further increase on the duration of boiling time to 30 min (method C), the total CNCbl content increased to 27.2 ng/g, but the obtained LC-MS/MS chromatogram still contained traces of unconverted cobalamins. Eventually, a second cyanidation step was introduced after paper filtration with shaking and light exposure (method D). With this method, the highest total CNCbl content of 29.9 ng/g was obtained. The corresponding LC-MS/MS chromatograms demonstrated complete conversions.

The optimized method (method D) was further tested on various meat samples including pork fillet, beef fillet, lamb fillet, chicken breast, and a pork liver sample. None of the obtained LC-MS/MS chromatograms showed residual peaks of unconverted cobalamins. Therefore, the optimized method was considered adequate for sample preparation and was further subjected to method validation.

### Method validation of the conversion SIDA method

#### LOD, LOQ, recoveries, and precisions of the conversion SIDA method

Table 2 summarizes LOD, LOQ, recoveries, and precisions of the conversion SIDA method for CNCbl as well as previous validation results of the native SIDA method for the same analyte.

**Table 2:** Method validation results of the conversion SIDA method in comparison to the native SIDA method [7]

Method	LOD (ng/g)	LOQ (ng/g)	Recovery (%)			Precisions (% RSD)		
			Level 1 (*n* = 3)	Level 2 (*n* = 3)	Level 3 (*n* = 3)	Inter-injection (*n* = 6)	Intra-day (*n* = 4)	Inter-day (*n* = 3)
Native [7]	0.19 [7]	0.68 [7]	98 ± 3 [7]	102 ± 2 [7]	101 ± 1 [7]	4 [7]	4 [7]	11 [7]
Conversion	0.09 ^*^	0.29 ^*^	111 ± 8	107 ± 6	101 ± 2	2	2	4

*, values determined based on surrogate analyte [^15^N_13_]-CNCbl

According to Vogelgesang and Hädrich [20], LODs and LOQs are determined from spiking experiments using a blank matrix. Previously, the soybean flour was used as the surrogate blank matrix in the validation of the native SIDA method without cyanidation [7] because it did not contain native cobalamins in detectable concentrations. However, when the conversion SIDA method was applied, the soybean flour showed trace amounts of CNCbl due to concentration of all cobalamins into CNCbl via cyanidation. Therefore, it could not be used as a blank matrix for validating the conversion SIDA method. Further investigation of various plant-derived matrices, including soy protein isolate, pea protein isolate, pumpkin seed protein isolate, rice protein isolate, almond protein isolate, and a self-prepared gluten sample from wheat flour, revealed ubiquitous presence of traces of cobalamins in foods. The levels of individual vitamers in the investigated matrices, which were too low to be detected by the native SIDA method, became detectable by the highly sensitive LC-MS/MS after all cobalamins were enriched as CNCbl by cyanidation conversion.

To tackle the issue of lacking absolute cobalamin-free blank matrices, the “surrogate analyte approach” [21] was adopted for determining the LOD and LOQ of the conversion SIDA method. The labeled compound [^15^N_13_]-CNCbl was spiked into the soybean flour in varying amounts as surrogate analyte, while the unlabeled CNCbl was added as internal standard for quantification. The soybean flour was used as the surrogate matrix as it demonstrated the lowest CNCbl signal among all investigated matrices when analyzed by the conversion SIDA method. With the application of the surrogate analyte approach, the LOD and LOQ were calculated from [^15^N_13_]-CNCbl instead of CNCbl based on the reverse LC-MS/MS response curve (details in the “LOD and LOQ” section). For quantification of [^15^N_13_]-CNCbl, the influence of trace CNCbl originating from the endogenous cobalamins in the soybean flour was negligible due to addition of high amounts of exogenous CNCbl as internal standard. The obtained LOD and LOQ were 0.09 and 0.29 ng/g, respectively, demonstrating high sensitivity of the conversion SIDA method for CNCbl analysis.

Recoveries of CNCbl were determined for the conversion SIDA method at three spiking levels of CNCbl in a pork fillet sample. The chosen spiking levels generally covered the concentration range of total cobalamins found in common meat samples. Applying the conversion SIDA method, good and reproducible recoveries ranging from 101 to 111% were obtained for CNCbl. Precisions were determined from analysis of a pork fillet sample applying the conversion SIDA method. As shown in Table 2, inter-injection, intra-day, and inter-day precisions were all below 4%.

When comparing the native and conversion SIDA methods based on the validation results for CNCbl, the performances of both methods were generally similar. The sensitivity and reproducibility of the conversion SIDA method for CNCbl were slightly better than that of the native SIDA method, respectively, reflected by the lower LOD/LOQ and the lower RSDs of the various precisions (Table 2). The better sensitivity of the conversion SIDA method for CNCbl might be due to the improved stability of the analyte with the addition of cyanide. The cyanidation conversion also acts as a concentrating step to enrich low levels of native cobalamins in samples during the extraction. The better reproducibility of the conversion SIDA method might be attributed to the higher concentrations of CNCbl being analyzed after pre-concentration, while much lower levels of native CNCbl present in the samples were determined by the native SIDA method. It is not feasible to compare the recoveries of the two methods directly as different matrices and spiking amounts were used for the two methods. The recoveries of the native SIDA method were determined by spiking a blank matrix at three levels of CNCbl (0.92, 5.71, and 9.13 ng/g) [7]. In contrast, the recoveries of the conversion SIDA method were determined by analyzing a pork fillet sample spiked with CNCbl at three different levels (7.5, 37.6, 74.7 ng/g) while accounting for the endogenous B12 content of the sample in the recovery calculation. Generally, both methods yielded satisfactory recoveries at their respective spiking levels.

In general, apart from that the conversion SIDA method showed slightly better sensitivity and precision, both the native and the conversion SIDA methods achieved similar method performances for CNCbl analysis in foods.

### Analysis of real meat samples by the conversion SIDA method

**Fig. 2:**
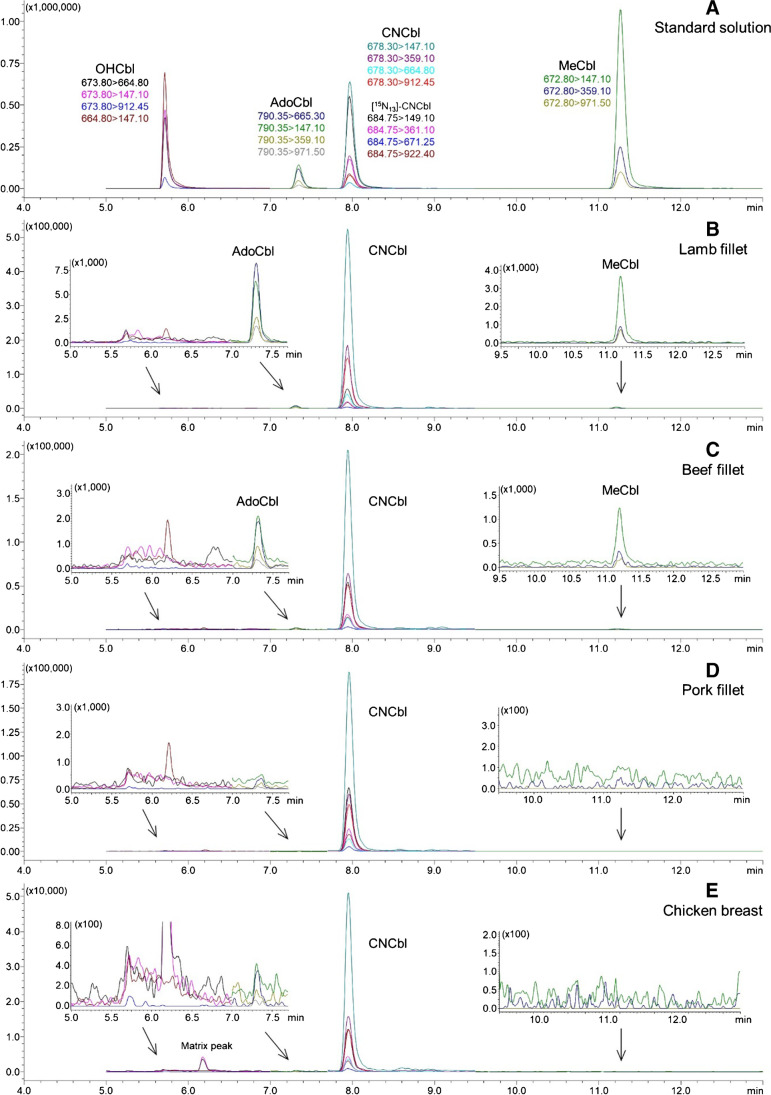
LC-MS/MS chromatograms of a mixed standard solution containing OHCbl, AdoCbl, CNCbl, MeCbl, and [^15^N_13_]-CNCbl (**A**) and meat samples using the validated conversion SIDA method (**B**–**E**)

The conversion SIDA method was employed to analyze real meat samples previously determined by the native SIDA method [7], following successful method validation. Surprisingly, results revealed underlying issues pertaining to the cyanidation conversion process. Notably, incomplete conversions were observed in selected samples, with the LC-MS/MS chromatograms of beef fillet and lamb fillet samples demonstrating residual peaks of OHCbl, AdoCbl, and MeCbl (Fig. 2). In contrast, the LC-MS/MS chromatograms of the pork fillet and chicken breast did not contain any unconverted cobalamin peaks. Earlier during the method development phase, the optimized sample preparation method (method D in Table 1) of the validated conversion SIDA method was tested on all meat samples, resulting in corresponding LC-MS/MS chromatograms without residual unconverted peaks (data not shown). Therefore, inconsistent results were obtained applying the same optimized sample preparation method between different days. These findings raised concerns regarding the inter-day reproducibility of the conversion SIDA method with respect to cyanidation conversion.

Several factors related to cyanidation conversion could contribute to the day-to-day variation of the method. First, the cyanidation conversion process utilizes light exposure and was performed under normal laboratory conditions. Consequently, factors that are challenging to regulate, such as the intensity and composition of natural light, could influence the process. Moreover, the sample matrices and cobalamin concentrations may as well play an important role in the performance of the cyanidation conversion, as residual peaks were observed in only certain meat samples. In addition, monitoring trace residual peaks of native cobalamins was intended for testing complete conversion. This approach strongly depends on the detection ability of the instrument, which may fluctuate day-to-day in the low concentration range. It must be noted that the LOD determined according to the approach of Vogelgesang and Hädrich [20] in this study accounts for such fluctuations and is actually higher than the real detection capability of the instrument. Finally, immunoaffinity columns, which are indispensable for analyte enrichment currently, can exhibit discrimination effects towards different vitamers [12], with batch-to-batch variation being another issue. Therefore, incomplete conversions in the samples will remain unseen in the LC-MS/MS chromatograms when the unconverted cobalamins are not properly pre-concentrated due to discrimination.

The conversion-associated issues could not be revealed during method validation as the spiking experiments used the target analyte CNCbl. The analysis of CNCbl does not require further cyanidation conversion. Therefore, although good validation results were obtained for CNCbl (details in the “Method validation of the conversion SIDA method” section), the conversion SIDA method developed and validated earlier cannot guarantee accurate determinations of total B12 contents in foods. The internal standard [^15^N_13_]-CNCbl used for SIDA quantification only compensates for the loss of CNCbl during sample treatment but not for the incomplete conversion of other cobalamins. However, this also applies to the previously reported conversion-based methods [12–14, 19], which were validated solely based on CNCbl without considering the conversion. Particularly in the cases of (U)HPLC-UV-based methods, monitoring unconverted cobalamins in trace concentrations may not be feasible due to the low sensitivity of the detection system, and this issue has not received adequate attention until now.

Regarding the quantitative results of meat samples determined by the conversion SIDA method, lower total B12 levels were obtained compared to the corresponding values from the previous study using the native SIDA method (Electronic Supplementary Material Table S3). The differences between these two sets of values could be attributed to a combination of factors, such as changes in cobalamins during sample storage and incomplete conversions when using the conversion SIDA method. In addition, native cobalamins may directly degrade during the extraction process instead of being converted to CNCbl. The internal standard [^15^N_13_]-CNCbl compensates for the degradation of CNCbl, but not for the degradation of unconverted cobalamins. Because the conversion-related problems were only discovered after the analysis, it is difficult to further determine the true causes. To gain further insight, the first step is to develop a more reliable method for monitoring conversion and to further optimize the cyanidation conversion process.

### Determination of the conversion rate of the current conversion SIDA method

**Fig. 3:**
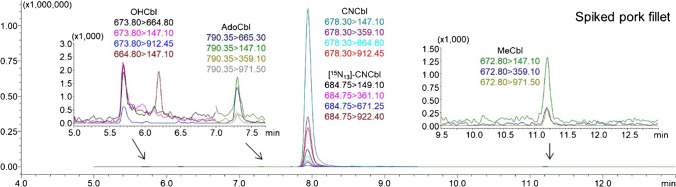
LC-MS/MS chromatogram of a spiked pork fillet sample applying the validated conversion SIDA method. The pork fillet sample was spiked with OHCbl (35.0 ng/g), AdoCbl (27.3 ng/g), MeCbl (39.5 ng/g), and [^15^N_13_]-CNCbl before cyanidation

To further gain insights into the conversion issue, a pork fillet sample was analyzed using the conversion SIDA method before and after being spiked with OHCbl (35.0 ng/g), AdoCbl (27.3 ng/g), and MeCbl (39.5 ng/g). The spiked pork fillet sample clearly demonstrated residual peaks of OHCbl, AdoCbl and MeCbl in the LC-MS/MS chromatograms (Fig. 3), whereas the original sample did not show any traces of unconverted cobalamins (Fig. 2). The obtained results suggest that either the conversion process in the pork fillet sample was dependent on the concentrations of cobalamins or the unconverted cobalamins in the original pork fillet sample could not be detected by the current monitoring method relying on immunoaffinity purification. A high conversion rate of 81 ± 2% (*n* = 3) was obtained using the equation presented in the “Determination of conversion rate” section. This suggests that, despite errors caused by conversion, the total B12 contents by quantification using the cyanidation conversion method may still be within an acceptable range. However, as the conversion rate was only determined in one type of meat with each cobalamin spiked at a single concentration, further conclusions about the associated errors with cyanidation await more investigations.

### Challenges for further optimization on cyanidation conversion-based methods

In this study, the use of LC-MS/MS is an improvement over previous methods that used less specific and sensitive analytical techniques. It allows the identification of unconverted cobalamins and provides more information relevant for evaluating the performance of the method. LC-MS/MS also represents state of the art for detecting trace compounds. In the field of vitamin analysis, cobalamins are the vitamins with the lowest concentrations in food and should be analyzed with LC-MS/MS, therefore. However, there are still reliability issues with this approach due to the intrinsic variation in instrument sensitivity at trace levels. Even when using LC-MS/MS, the use of an immunoaffinity column for sample preparation is still essential. Therefore, without a further breakthrough in improving the sensitivity of LC-MS/MS at the instrumental level, the potential discriminatory effects towards different vitamers could not be further resolved as long as immunoaffinity purification is still required. It should be noted that previous (U)HPLC-UV-based methods, which also have to rely on immunoaffinity purification, are also affected by the potential discriminatory effects.

A reliable monitoring method is a prerequisite for further evaluation of the performance of cyanidation conversion, and such a method ultimately requires the elimination of immunoaffinity columns for sample preparation. However, this could only be achieved by using more sensitive analytical systems than the instruments that are currently available on the market. This may be possible in the near future with the next generation of triple quadrupole mass spectrometers.

## Conclusion

The application of cyanidation conversion in combination with SIDA, i.e., the conversion SIDA method, was employed to investigate its efficacy for total B12 determination. The developed and validated conversion SIDA method demonstrated low LOD and LOQ, good recoveries, and high precision for CNCbl analysis. Comparative evaluation of the conversion SIDA method and the native SIDA method showed similar performance in terms of CNCbl validation results. However, inconsistencies were observed in the completeness of cyanidation conversion between results obtained during the method development stage and those obtained during subsequent application to real meat samples, highlighting the day-to-day variability and reliability challenges associated with the conversion SIDA method.

Notably, the limitations of the conversion SIDA method were not apparent during method validation as spiking experiments were performed with the target analyte CNCbl. To address these limitations, it is crucial to develop a more reliable monitoring method that overcomes constraints related to instrument sensitivity, stability, and the discriminatory effects of immunoaffinity purification on different vitamers. In addition, the optimization of the cyanidation conversion process is essential. However, advances in analytical techniques are needed to overcome current limitations.

While previous B12 analytical methods commonly employed cyanidation conversion to reduce analyte complexity, increase analyte concentrations, and improve analyte stability, the present study revealed limitations associated with this approach. Future studies intending to employ cyanidation conversion should focus on achieving complete and reproducible conversions for B12 quantification. Moreover, regardless of the analytical system in use, routine verification of quantitative results from cyanidation-based methods is recommended.

## Supplementary Information

ESM 1 (DOCX 32 kb)

## References

[1] Kolhouse JF, Allen RH (1977). Absorption, plasma transport, and cellular retention of cobalamin analogues in the rabbit: evidence for the existence of multiple mechanisms that prevent the absorption and tissue dissemination of naturally occurring cobalamin analogues. J Clin Invest..

[2] Ludwig ML, Matthews RG (1997). Structure-based perspectives on B12-dependent enzymes. Annu Rev Biochem..

[3] Froese DS, Fowler B, Baumgartner MR (2019). Vitamin B12, folate, and the methionine remethylation cycle-biochemistry, pathways, and regulation. J Inherited Metab Dis..

[4] Lyon P, Strippoli V, Fang B, Cimmino L (2020). B vitamins and one-carbon metabolism: implications in human health and disease. Nutrients..

[5] Roth JR, Lawrence JG, Bobik TA (1996). Cobalamin (coenzyme B12): synthesis and biological significance. Annu Rev Microbiol..

[6] Allen RH, Stabler SP (2008). Identification and quantitation of cobalamin and cobalamin analogues in human feces. Am J Clin Nutr..

[7] Wang M, Asam S, Chen J, Rychlik M (2021). Development of stable isotope dilution assays for the analysis of natural forms of vitamin B12 in meat. J Agric Food Chem..

[8] Wang M, Asam S, Chen J, Ehrmann M, Rychlik M (2021). Production of four 15N-labelled cobalamins via biosynthesis using Propionibacterium freudenreichii. Front Microbiol..

[9] Kelleher BP, Broin SD (1991). Microbiological assay for vitamin B12 performed in 96-well microtitre plates. J Clin Pathol..

[10] Eitenmiller RR, Landen WO, Jeon IJ, Ilkins WG (1995). Vitamins. Analyzing food for nutritional labeling and hazardous contaminants.

[11] Heudi O, Kilinc T, Fontannaz P, Marley E (2006). Determination of vitamin B12 in food products and in premixes by reversed-phase high performance liquid chromatography and immunoaffinity extraction. J Chromatogr..

[12] Marley E (2009). Characterisation of vitamin B12 immunoaffinity columns and method development for determination of vitamin B12 in a range of foods, juices and pharmaceutical products using immunoaffinity clean-up and high performance liquid chromatography with UV detection. Food Addit Contam Part A..

[13] Guggisberg D, Risse MC, Hadorn R (2012). Determination of vitamin B12 in meat products by RP-HPLC after enrichment and purification on an immunoaffinity column. Meat Sci..

[14] Chamlagain B, Edelmann M, Kariluoto S, Ollilainen V, Piironen V (2015). Ultra-high performance liquid chromatographic and mass spectrometric analysis of active vitamin B12 in cells of Propionibacterium and fermented cereal matrices. Food Chem..

[15] Campos-Giménez E, Martin F, Collaborators. (2018). Vitamin B12 (cyanocobalamin) in infant formula adult/pediatric nutritional formula by liquid chromatography with ultraviolet detection: collaborative study, final action 2014.02. J AOAC Int..

[16] Schmidt A, Call LM, Macheiner L, Mayer HK (2019). Determination of vitamin B12 in four edible insect species by immunoaffinity and ultra-high performance liquid chromatography. Food Chem..

[17] Nakos M, Pepelanova I, Beutel S, Krings U, Berger RG, Scheper T (2017). Isolation and analysis of vitamin B12 from plant samples. Food Chem..

[18] Dubascoux S, Richoz Payot J, Sylvain P, Nicolas M, Campos GE (2021). Vitamin B12 quantification in human milk – beyond current limitations using liquid chromatography and inductively coupled plasma – mass spectrometry. Food Chem..

[19] D'Ulivo L, Yang L, Ding JF, Pagliano E, Leek DM, Thibeault MP (2017). Determination of cyanocobalamin by isotope dilution LC-MS/MS. Anal Chim Acta..

[20] Vogelgesang J, Hadrich J (1998). Limits of detection, identification and determination: a statistical approach for practitioners. Accredit Qual Assur..

[21] Jones BR, Schultz GA, Eckstein JA, Ackermann BL (2012). Surrogate matrix and surrogate analyte approaches for definitive quantitation of endogenous biomolecules. Bioanalysis..

[22] Mandel J (1964). The statistical analysis of experimental data.

[23] Cobalamins LJ, Leenher AP, Lambert WE, Bocxlaer JF (2000). Modern chromatographic analysis of vitamins.

[24] Muhammad K, Briggs D, Jones G (1993). The appropriateness of using cyanocobalamin as calibration standards in Lactobacillus leichmannii ATCC 7830 assay of vitamin B12. Food Chem..

