# Supplementary material for: Challenges in the determination of total vitamin B12 by cyanidation conversion: insights from stable isotope dilution assays
Source: Anal Bioanal Chem. 2023 Jul 19;415(23):5797–807. doi: 10.1007/s00216-023-04860-y (PMC10474169; doi:10.1007/s00216-023-04860-y)
Supplement: Supplementary file 1 — (DOCX 32 kb) [file 216_2023_4860_MOESM1_ESM.docx]

Supporting information

**Challenges in the determination of total vitamin B12 by cyanidation conversion: Insights from stable isotope dilution assays.**

Mengle Wang*, Kathrin Schuster, Stefan Asam, Michael Rychlik

Chair of Analytical Food Chemistry, Technical University of Munich, 85354 Freising, Germany

*Corresponding author:

Postal address: Maximus-von-Imhof-Forum 2, 85354 Freising, Germany

Fax: + 49 8161712297

Email: [mengle.wang@tum.de](mailto:mengle.wang@tum.de)

Table S1. HPLC-DAD response functions for OHCbl, AdoCbl and MeCbl [1]

| **Analyte** | ***λ* (nm)** | **HPLC-DAD response curve ^a^** | ***R^2^*** |
| --- | --- | --- | --- |
| OHCbl | 351 | *y* = 1.1518*x* - 0.0428 | 0.9998 |
| AdoCbl | 377 | *y* = 0.8036*x* - 0.0055 | 0.9999 |
| MeCbl | 341 | *y* = 1.0648*x* - 0.0137 | 0.9999 |
| Note: ^a^, y are peak area ratios [A(Analyte)/A(CNCbl)] and x are mass concentration ratios [ρ(Analyte)/ρ(CNCbl)]; CNCbl as internal standard, concentration pre-determined by UV spectroscopy at 361 nm. | | | |

**Table S2.** LC-MS/MS MRM parameters of CNCbl, [^15^N_13_]-CNCbl, OHCbl, AdoCbl and MeCbl [1]

| **Analyte** | **Precursor**  **(m/z)** | **Product**  **(m/z)** | **Dwell time**  **(ms)** | **Q1 Pre-bias**  **(V)** | **CE**  **(V)** | **Q3 Pre-bias**  **(V)** | **Acquisition time**  **(min)** |
| --- | --- | --- | --- | --- | --- | --- | --- |
| CNCbl | 678.30 | **147.10^1^** | 70.0 | 34.0 | 37.0 | 28.0 | 7.7 - 9.5 |
|  |  | 359.10^1^ | 70.0 | 34.0 | 24.0 | 14.0 |  |
|  |  | 912.45^1^ | 70.0 | 20.0 | 34.0 | 28.0 |  |
| [^15^N_13_]-CNCbl | 684.75 | **149.10^1^** | 70.0 | 34.0 | 37.0 | 28.0 | 7.7 - 9.5 |
|  |  | 361.10^1^ | 70.0 | 34.0 | 24.0 | 14.0 |  |
|  |  | 922.40^1^ | 70.0 | 20.0 | 34.0 | 28.0 |  |
| OHCbl | 673.80 | 664.80^2^ | 70.0 | 36.0 | 9.0 | 20.0 | 5.0 - 7.0 |
|  |  | 147.10^1^ | 70.0 | 20.0 | 51.0 | 28.0 |  |
|  |  | 912.45^1^ | 70.0 | 20.0 | 31.0 | 28.0 |  |
|  | 664.80* | 147.10^1^ | 70.0 | 34.0 | 50.0 | 30.0 |  |
| AdoCbl | 790.35 | 665.30^2^ | 70.0 | 22.0 | 21.0 | 26.0 | 7.0 - 7.7 |
|  |  | 147.10^1^ | 70.0 | 22.0 | 49.0 | 26.0 |  |
|  |  | 359.10^1^ | 70.0 | 22.0 | 30.0 | 26.0 |  |
|  |  | 971.50^1^ | 70.0 | 24.0 | 33.0 | 38.0 |  |
| MeCbl | 672.80 | 147.10^1^ | 100.0 | 20.0 | 47.0 | 28.0 | 9.5 - 13.0 |
|  |  | 359.10^1^ | 100.0 | 36.0 | 28.0 | 26.0 |  |
|  |  | 971.50^1^ | 100.0 | 20.0 | 30.0 | 38.0 |  |

*Note: all precursor ions are double charged; product ions indicated with superscripts “1” and “2” are single and double charged, respectively. The first transitions of CNCbl and [^15^N_13_]-CNCbl (highlighted in bold) were used for quantitation of total CNCbl. * Precursor ions formed with the cleavage of upper ligand (i.e. -OH group) during ionization.*

**Table S3.** Total CNCbl contents of meat samples determined applying the conversion SIDA method in comparison to previous values determined by the native SIDA method.

| **Sample** | **Conversion SIDA method (ng/g)** | | **Native SIDA method* (ng/g)** |
| --- | --- | --- | --- |
|  | **Individual extraction^#^** | **Mean ± SD** |  |
| Pork fillet | 16.92 ± 0.20 | 16.93 ± 0.25 (n = 4) | 22.92 ± 1.33 (n = 12) ^[1]^ |
|  | 16.74 ± 0.16 |  |  |
|  | 16.98 ± 0.09 |  |  |
|  | 17.09 ± 0.42 |  |  |
| Beef fillet | 21.49 ± 0.04 | 21.91 ± 1.04 (n = 3) | 30.45 ± 0.08 (n = 3) ^[1]^ |
|  | 22.95 ± 1.36 |  |  |
|  | 21.30 ± 0.07 |  |  |
| Lamb fillet | 52.30 ± 0.44 | 52.66 ± 0.97 (n = 3) | 71.79 ± 2.93 (n = 4) ^[1]^ |
|  | 52.99 ± 1.55 |  |  |
|  | 52.68 ± 0.88 |  |  |
| Chicken breast | 4.73 ± 0.16 | 4.72 ± 0.48 (n = 3) | 9.44 ± 1.04 (n = 4) ^[1]^ |
|  | 4.62 ± 0.85 |  |  |
|  | 4.79 ± 0.38 |  |  |
| Note: SD, standard deviation; ^#^, results presented as mean ± SD from triplicate injections; *, total B12 calculated as CNCbl equivalent from previously determined individual cobalamin concentrations obtained previously by the native SIDA method [1]. | | | |

**Reference:**

1. Wang M, Asam S, Chen J, Rychlik M. Development of Stable Isotope Dilution Assays for the Analysis of Natural Forms of Vitamin B12 in Meat. J Agric Food Chem. 2021;69(36):10722-30.
